# Impact of Cardiac Interoception on the Self-Prioritization Effect

**DOI:** 10.3389/fpsyg.2022.825370

**Published:** 2022-07-12

**Authors:** Tatsuru Honda, Takashi Nakao

**Affiliations:** ^1^Graduate School of Education, Hiroshima University, Hiroshima, Japan; ^2^Graduate School of Humanities and Social Sciences, Hiroshima University, Hiroshima, Japan

**Keywords:** self-prioritization effect, interoception, interoceptive accuracy, cardiac cycle, self-relevance

## Abstract

Self-relevant information is processed faster and more accurately than non-self-relevant information. Such a bias is developed even for newly associated information with the self, which is also known as the self-prioritization effect (SPE). Interoception, which refers to the overall processing of information from within the body, is crucial for self-relevant processing; however, its role in SPE remains unexplored. In this study, we investigated the relationship between the magnitude of SPE and interoceptive accuracy (IAc), defined as one’s ability to accurately perceive one’s own interoceptive state. Additionally, to explore the causal relationship, we measured SPE by presenting self- or other-relevant stimuli based on the participant’s cardiac cycle in the shape-label matching task. We demonstrated that IAc was negatively correlated with the magnitude of SPE in terms of discrimination of the relevance of the stimuli. In addition, a correlation was observed only when the stimuli were presented during cardiac systole. Furthermore, IAc was negatively correlated with the processing of self-relevant stimuli but not with other-relevant stimuli. Collectively, our results show that individuals with higher IAc have relatively lower discriminative sensitivity to newly and temporary associated self-relevant stimuli presented during the accentuation of cardiac interoceptive information. Although SPE is a phenomenon in which newly self-associated stimuli are preferentially processed, our results suggest that individuals with higher IAc prioritized processing interoceptive information over temporarily associated self-relevant external information. Conversely, previous studies using paradigms other than the shape-label matching paradigm with familiar self-relevant stimuli, such as self-face, reported that interoceptive information enhances the processing of self-relevant stimuli. Whether interoceptive information enhances the processing of external self-relevant information may depend on the familiarity with the self-relevant stimuli and the experimental paradigm.

## Introduction

The self has attracted the interest of researchers in psychology for a long time. In the field of experimental psychology, researchers have attempted to understand the self from a functional perspective by quantifying the self-bias effect (Sui and Humphreys, 2015; Sui and Gu, 2017). For example, it is well known that people can be aware of their own name being called, even in situations where they are not paying attention—known as the cocktail party effect (Moray, 1959). Information related to the self, such as one’s own name, attracts more attention than non-self-relevant stimuli. Furthermore, biases have been demonstrated in a variety of external stimuli and cognitive processes (Sui and Humphreys, 2015; Cunningham and Turk, 2017).

Recently, a novel paradigm has demonstrated that biases emerge even for neutral stimuli that are newly associated with one’s self. In the shape-label matching paradigm (Sui et al., 2012), participants were first asked to learn the associations between geometric shapes and personal labels (e.g., circles represent self, triangles represent best friends, and squares represent strangers). Following the learning phase, the participants judged whether the presented shape-label parings matched the previously learned associations. The results showed that their responses to self-relevant stimuli (i.e., circle–self) were faster and more accurate than non-self-relevant stimuli. This is known as the self-prioritization effect (SPE). This paradigm demonstrated that individuals rapidly develop biases, even for newly associated stimuli with the self. Furthermore, although self-relevance and intimacy cannot be separated when using familiar stimuli such as one’s own name or face, which have been widely used in conventional studies, the shape-label matching paradigm allows us to examine self-bias without the influence of familiarity (Kim and Florack, 2021). SPE has been observed in the shape-label matching paradigm for various types of stimuli (Sui et al., 2012; Payne et al., 2017; Sel et al., 2019) and other sensory modalities (Frings and Wentura, 2014; Schäfer et al., 2016).

Although SPE is observed when external stimuli are associated with the current self, no such preferential processing is observed when associated with the past or the future self (Golubickis et al., 2017; Kim and Florack, 2021). Golubickis et al. (2017) examined whether SPE could emerge when associating a geometric shape with a temporally distant self (i.e., current self vs. future self) in the shape-label matching paradigm. The results showed that SPE did not emerge in the stimuli associated with the temporally distant self; rather, it presented with the current self alone. These results can be explained by the fact that we sometimes treat our temporally distant selves as strangers (Pronin and Ross, 2006; Pronin et al., 2008). Considering that such “self-becomes-other” effects are caused by the lack of information on internal states (Pronin and Ross, 2006; Pronin et al., 2008) that information may play a crucial role in the emergence of SPE.

Interoception is one of the internal states of the self. It refers to the overall process of how the nervous system senses, interprets, and integrates information arising from within the body, providing a moment-by-moment representation of internal bodily states with or without awareness (Berntson and Khalsa, 2021). Interoception supports homeostatic control, allows allostatic adaptation, and drives behavior through feelings such as hunger and thirst (Tsakiris and Critchley, 2016). Several theories have proposed that interoception is the foundation of the self (Craig, 2009; Seth, 2013), and previous empirical findings support these proposals. For instance, heartbeat-evoked responses within the default mode network (Baranauskas et al., 2021) covaried with self-relatedness of spontaneous thoughts (Babo-Rebelo et al., 2016). Qin et al. (2020) conducted a meta-analysis of neuroimaging studies and concluded that interoceptive information is necessary for processing self-relevant information. Furthermore, Ambrosini et al. (2019) reported that cardiac interoceptive information speeds up recognizing one’s own face. Moreover, observation of a fake body pulsating in synchrony with one’s heartbeat induces the feeling that a fake body is one’s own (i.e., a sense of body-ownership; Aspell et al., 2013; Suzuki et al., 2013; Heydrich et al., 2018, 2021). These findings revealed that interoception contributes to self-relevant processing. Thus, on considering the findings on interoception and SPE, it is indicated that SPE emerges when external stimuli are associated with the current self, driven by interoception. In other words, it is possible that the more interoceptive information available, the stronger SPE emerges.

However, we could assume an opposite relationship between SPE and interoception as well. Several reports have shown that individuals who have greater availability of their own interoceptive information are less likely to recognize external stimuli as being associated with themselves. Schauder et al. (2015) and Tsakiris et al. (2011) used the classical rubber-hand illusion paradigm with visuo-tactile stimulation (Botvinick and Cohen, 1998). They demonstrated that individuals with a greater ability to accurately perceive their own interoceptive state (interoceptive accuracy: IAc; Garfinkel et al., 2015) are less susceptible to the illusion involving the fake hands. A similar finding has been reported in the enfacement illusion paradigm, in which participants recognize others’ faces as their own by seeing another person’s face being stroked in synchrony with their own (Tajadura-Jiménez and Tsakiris, 2014). These studies showed that individuals with higher IAc are less likely to recognize external bodily stimuli as part of their own bodies. Although the typical shape-label matching paradigm using the geometric shapes investigates the association of the self with a geometric shape rather than bodily stimuli, these findings allow us to predict that individuals with higher IAc show weaker SPE.

Thus, there are two possibilities for the relationship between SPE and interoception: interoception either promotes or inhibits SPE. However, to the best of our knowledge, the relationship between SPE and interoception has not yet been investigated.

The present study aimed to elucidate the relationship between SPE and interoception. As mentioned above, a positive relationship between SPE and interoception is predicted from studies of the shape-label matching paradigm and interoception. In the shape-label matching paradigm, it has been reported that stimuli associated with a temporally distant self can dampen the emergence of SPE and do not facilitate information processing, differing from stimuli associated with the current self (Golubickis et al., 2017; Kim and Florack, 2021). This may be because a self that is temporally distant from its current self lacks access to and availability of information about the internal state, that is, interoceptive information (Pronin and Ross, 2006). Therefore, individuals with higher IAc, who can perceive their interoceptive information accurately, are predicted to show a stronger SPE.

Conversely, we can predict a negative relationship between SPE and IAc as well. In the classic rubber-hand illusion paradigms with visuo-tactile stimulation, individuals with higher IAc are less susceptible to the illusion over the fake hands (Tsakiris et al., 2011; Schauder et al., 2015). Similarly, in the enfacement illusion paradigm, individuals with higher IAc have been reported to be less incorporating of other’s faces into their own facial representations (Tajadura-Jiménez and Tsakiris, 2014). Therefore, assuming that the same relationship applies to the association between the self and the external geometric shapes, individuals with higher IAc are predicted to show weaker SPE in the shape-label matching paradigm.

Furthermore, to get closer to an understanding of the causal relationship between interoception and SPE, we manipulated the timing of stimuli presentations based on the cardiac cycle. Cardiac afferent information (i.e., strength and timing of individual heartbeats) is transmitted to the brain through arterial baroreceptors. The receptors fire during systole in the cardiac cycle and cardiac afferent information is accentuated, while they are quiescent during diastole (Garfinkel and Critchley, 2016; Critchley and Garfinkel, 2018). Therefore, previous studies have investigated the impact of interoception on cognitive processing by presenting stimuli coincident with systole or diastole, employing the phasic nature of cardiac afferent information (e.g., Gray et al., 2012; Garfinkel et al., 2014). These studies have reported that stimuli presentations during systole modulate cognitive processing, including self-face recognition (Ambrosini et al., 2019). Considering that individuals with higher IAc are better at receiving interoceptive information, such interoceptive impact may be stronger as well. Indeed, von Mohr et al. (2021) observed an interaction between cardiac interoceptive impact and IAc, measuring the heartbeat counting task (Schandry, 1981) on emotional egocentricity bias. They showed that individuals with higher IAc are more affected by their own cardiac information. Taking these findings into account, if interoceptive information impacts the emergence of SPE, the following results are predicted: The correlation between IAc and SPE is predicted when the stimuli are presented coincident with systole because the cardiac afferent information is accentuated during this phase and individuals with higher IAc are more sensitive to cardiac afferent information. In contrast, the correlation would not be observed when the stimuli are presented during diastole because baroreceptors are quiescent and less affected by interoceptive impacts regardless of whether IAc is high or low.

In summary, we hypothesized that (1) IAc is positively or negatively correlated with SPE and (2) the correlation is observed only under conditions where the stimuli are presented during systole in the cardiac cycle.

## Materials and Methods

### Participants

The participants included 38 Japanese university students. Two participants were excluded due to hardware failure or low-quality of recorded ECG data. Thus, a total of 36 participants (18 women, aged 20.6 ± 1.9 years) were included in the final analyses. The participants had normal or corrected-to-normal visual acuity.

The protocol was reviewed and approved by the Ethical Committee of the Graduate School of Education, Hiroshima University. All participants signed an informed consent form and were instructed that they could discontinue participating in the experiment at any time. The participants received 2,000 yen as compensation after finishing the experiment.

### Materials and Apparatus

The experiment was programmed, and all data were recorded using MATLAB 2019b software (Mathworks). Stimuli were presented using a 24-inch monitor (1,920 × 1,080 at 60 Hz) placed approximately 60 cm from the participant. ECG was continuously recorded to measure the heart activity of the participants. Three Ag/AgCl electrodes were placed in lead II configuration: Two electrodes were positioned underneath the left and right collarbone and the other on the lower left rib of each participant. The ECG signal was recorded at 400 Hz with a BIOPAC MP160 and a BN-RSPEC amplifier (BIOPAC System, United States of America) using MATLAB 2019b software.

### Procedure

All participants were tested individually in a shielded room. The participants were instructed about the experimental procedure and signed a consent form. After the placement of the ECG electrodes, the participants were instructed to sit on a comfortable chair. Subsequently, the participants engaged in two tasks: One was a shape-label matching task with presentation timing manipulated based on the cardiac cycles and the other was a heartbeat counting task (Schandry, 1981).

#### Perceptual Matching Task

We conducted a shape-label matching task to quantify individuals’ SPE. This task consisted of two phases: a learning and a matching phase. In the learning phase, participants were predicted to learn three associations between geometrical shapes and social labels (e.g., circle–self, triangle–best friend, and square–stranger) in 60 s. The shapes were described linguistically and were not presented as images during this phase.

**Figure 1:**
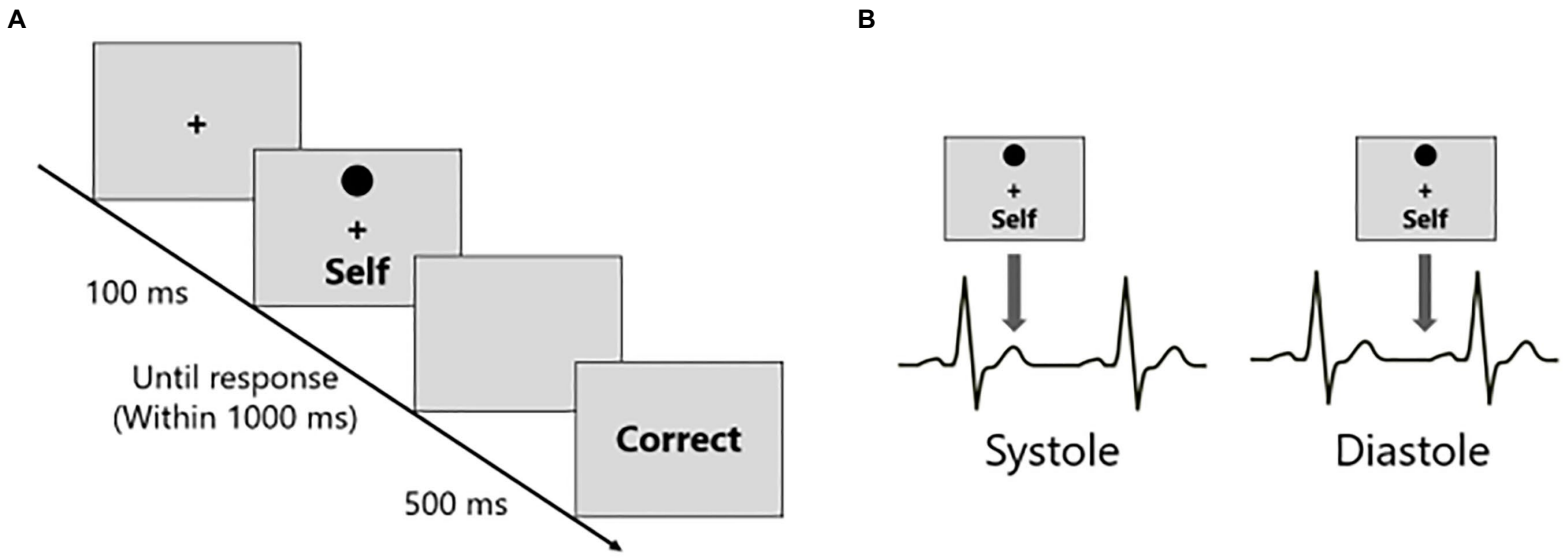
The procedures of the matching phase and the timings of stimuli presentations in the shape-label matching task. **(A)** The participants judged whether the presented shape-label pair was matched based on the associations learned during the learning phase. **(B)** The shape-label pairs were presented in coincidence with either systole (T-wave peak) or diastole (T-wave peak + 250 ms) in the participant’s cardiac cycle.

After the learning phase, a matching phase was conducted (Figure 1A). Participants were required to judge whether the presented shape-label pairs were matched based on the associations learned during the learning phase. Additionally, to examine the relationship between SPE and interoception, the stimuli were presented either during systole or diastole. Corresponding to the previous studies (Gray et al., 2012; Garfinkel et al., 2014), in this experiment, stimuli were presented at the T-wave peaks in the Systole, and at 250 ms post-T-wave peaks in the Diastole conditions, respectively (Figure 1B). The T-wave peaks were detected in real-time when the amplitude of the ECG signal exceeded a predefined threshold. The threshold was determined by the experimenter based on the resting state of the heart for 15 s before beginning the experimental task.

In the matching phase, each trial began with the presentation of the fixation cross, the duration of which was determined by the detection of the T-wave peaks. The mean time of the fixation cross was 1323.66 ms (SD = 106.65 ms). Subsequently, a shape and a label were presented above and below the fixation cross for 100 ms, respectively, followed by a blank screen. The presentation of the shape-label pair was time-locked to either the T-wave peaks (systole condition) or delayed 250 ms from the T-wave peaks (diastole condition). Within the blank screen, participants were required to judge whether the presented shape-label pairing was matched by pressing one of the two buttons (J key or F key on the keyboard) as quickly and accurately as possible, based on the previously learned associations. The corresponding of the J key or the F key to the matching or non-matching response was counterbalanced across the participants. Feedback was given (“CORRECT,” “INCORRECT,” and “LATE”) for 500 ms according to the participant’s response at the end of each trial. If the participant did not respond within 1,000 ms, it was defined as “LATE.” Participants conducted 30 trials for each of the 12 conditions (self-matching at systole, self-nonmatching at systole, friend-matching at systole, friend-nonmatching at systole, stranger-matching at systole, stranger-nonmatching at systole, and similar combinations at diastole). There were five blocks of 72 trials each. In each block, six trials for each of the 12 conditions were conducted in a pseudo-randomized order. All stimuli were colored black and presented on a gray background.

#### Heartbeat Counting Task

Individual differences in IAc were quantified using a heartbeat counting task. Participants were asked to silently count only their perceived heartbeats without taking their pulse (Schandry, 1981). Additionally, considering that the knowledge of heart rate can affect performance, they were told not to guess the heartbeats based on their knowledge (Desmedt et al., 2018). Each trial began with a warning tone at 800 Hz, and approximately 3 s later, a 1,000 Hz tone was given indicating the start of the counting interval, and participants began counting their heartbeats. Finally, a 1,000 Hz tone was sounded to indicate the end of the counting interval, and the participants reported their perceived number of heartbeats. There were six trials with variable time windows of 25, 30, 35, 40, 45, and 50 s for the counting intervals. The trials were presented in a randomized order. Moreover, the participants received no information about the length of each interval.

### Data Analysis

#### Shape-Label Matching Task

We assessed reaction time (RT) and discrimination sensitivity (d-prime) as indices of SPE. Only correct responses in matching trials were included in the RT analysis. The d-prime refers to how discriminable the signal (i.e., match trials) is from the background noise (i.e., mismatch trials), and is calculated based on the signal detection approach. In the case where hit rates and false alarm rates are 1.0 or 0, the corresponding z-scores are infinite and d-prime cannot be calculated. Therefore, we corrected all hit rates and false alarm rates by adding 0.5 to the number of hits and false alarms and dividing by *N* + 1, where *N* is the number of “match” or “mismatch” trials (Snodgrass and Corwin, 1988). Subsequently, we also assessed the magnitude of SPE (i.e., the magnitude to which self-relevant stimuli are processed faster or more accurately than other-relevant stimuli) for each individual for both RT and d-prime. The magnitude of SPE on RT was calculated by the difference between the Stranger and Self conditions, divided by the sum of the two conditions [that is, (stranger – self)/(self + stranger)]. The magnitude of SPE on d-prime was calculated by the difference between the self and stranger conditions, divided by the sum of the two conditions [that is, (self – stranger)/(self + stranger)]. Higher values for both indicated that self-relevant stimuli were processed more preferentially than other-relevant stimuli. The reason for including the Friend condition that was not related to assessing the magnitude of SPE was to make the task sufficiently challenging (Verplanken and Sui, 2019). The accuracy rate for the typical shape-label matching task with three conditions (i.e., self, friend, and stranger conditions) is reported to be high (Sui et al., 2012, Exp. 1). Therefore, if the task was conducted under only two conditions, excluding the Friend condition, there was a risk of ceiling effects caused by further improving the performance of the task. To avoid this, we included the Friend condition as in many previous studies (e.g., Sui et al., 2012; Payne et al., 2017).

#### Heartbeat Counting Task

Interoceptive accuracy was assessed according to the following formula: (1/6)*Σ[1–(|ECG recorded heartbeats – participant counted heartbeats|/ECG recorded heartbeats)] (Schandry, 1981). IAc scores range from 0 to 1, with higher scores indicating better IAc.

## Results

### Validation of Stimuli Onset Times

First, in the shape-label matching task, we examined whether the stimuli were accurately presented during systole or diastole. This is because the timing of stimuli presentation was based on the participants’ T-wave peaks, but the detection criteria of T-wave peaks were set by the experimenter for each participant to detect T-wave peaks in real-time, which may have differed from the actual T-wave peaks. Therefore, T-wave peaks (actual T-wave peaks) were determined *a posteriori* from the recorded ECG waveforms of each participant during the shape-label matching task, and the temporal difference from the T-wave peaks set during the task (established T-wave peaks) was calculated (actual—established). The results showed that the temporal difference was 18.32 ms (SD = 7.28 ms), indicating that the T-wave peaks were detected almost accurately during the experimental task. The stimuli in the Systole and Diastole conditions were presented at T-wave peaks – 11.92 ms (SD = 7.55 ms) and T-wave peaks + 243.72 ms (SD = 7.11 ms), respectively, which were close to the target timing. Furthermore, we confirmed that all stimuli were within the R-R interval at which the T-wave peaks were detected as a criterion. These results indicated that the timing of the stimuli presentation was appropriate in the shape-label matching task.

### Self-Prioritization Effect in the Shape-Label Matching Task

Table 1 summarizes the mean RT and d-prime of each condition. To confirm the emergence of SPE in the shape-label matching task, we conducted a 3 (Label) × 2 (Cardiac cycle) repeated-measures analysis of variance for both RTs and d-prime. For RT, the result showed a significant main effect of the Label (*F*(2, 70) = 18.01, *p* < 0.01, *η_p_*^2^ = 0.34), shorter RT in the Self condition than either the Friend or Stranger conditions (both *p* < 0.01), and shorter RT in the Friend condition than in the Stranger condition (*p* < 0.01). We did not find any significant difference in the Cardiac cycle factor (*F*(1, 35) = 0.81, *p* = 0.38, *η_p_*^2^ = 0.02) and interaction effects (*F*(2, 70) = 1.18, *p* = 0.31, *η_p_*^2^ = 0.03). For d-prime, the result showed a significant main effect of the Label (*F*(2, 70) = 4.59, *p* = 0.01, *ηp*^2^ = 0.12), with larger d-prime in the Self condition than the Stranger condition (*p* = 0.01), and with no difference between the Self and Friend or Friend and Stranger conditions. No significant difference was found in the Cardiac cycle factor (*F*(1, 35) = 3.25, *p* = 0.08, *η_p_*^2^ = 0.09) and interaction effects (*F*(2, 70) = 0.52, *p* = 0.60, *η_p_*^2^ = 0.02). These results indicate that SPE emerged in the shape-label matching task.

**Table 1:** Mean reaction time (RT) and d-prime as a function of label category and cardiac cycle.

Cardiac cycle	Label category	Mean RT (ms)		d-prime	
Both (systole and diastole)	Self	561.13	(66.30)	3.12	(0.80)
	Friend	596.08	(64.45)	2.83	(0.82)
	Stranger	624.58	(62.88)	2.64	(0.67)
Systole	Self	559.71	(64.97)	2.99	(0.69)
	Friend	599.71	(65.46)	2.70	(0.80)
	Stranger	626.19	(66.34)	2.53	(0.65)
Diastole	Self	562.48	(70.19)	3.02	(0.83)
	Friend	591.95	(66.92)	2.88	(0.92)
	Stranger	623.14	(63.72)	2.68	(0.77)

*Standard deviations (SDs) are shown in parentheses*.

### Correlation Analyses

**Figure 2:**
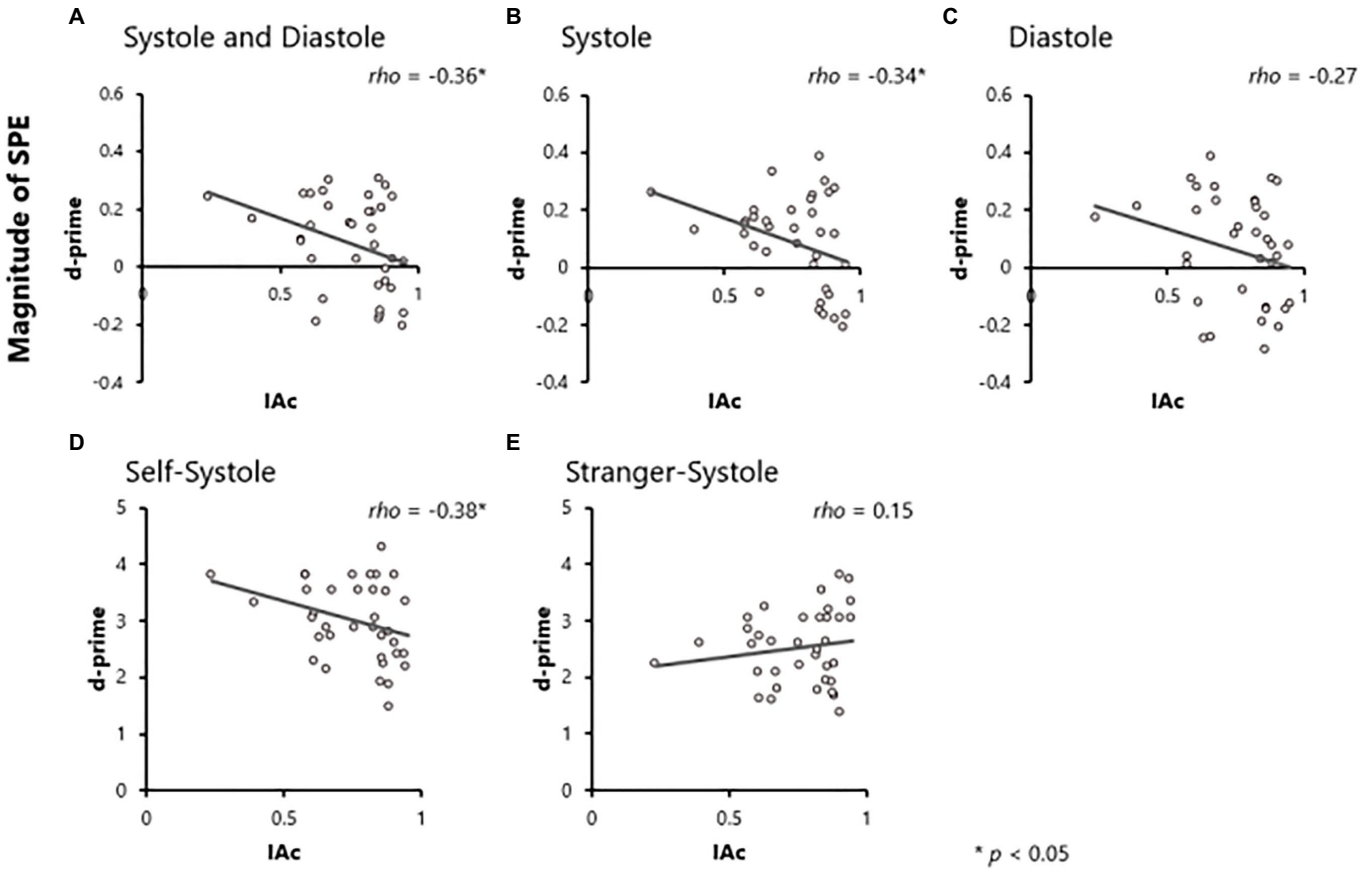
Results of correlation analyses. Correlations between IAc and the magnitude of self-prioritization effect (SPE) on d-prime in the both systole and diastole **(A)**, systole **(B)** and diastole **(C)** conditions. Correlations between interoceptive accuracy (IAc) and d-prime in the self-systole **(D)** and stranger-systole **(E)** conditions.

Correlation analyses were conducted to explore the relationship between the magnitude of SPE on both RT and d-prime, and IAc, respectively (Hypothesis 1). We used Spearman rank correlation analysis because IAc scores were not normally distributed. The results revealed that there was a significant negative correlation between the magnitude of SPE on d-prime and IAc (*rho* = −0.36, *p* = 0.03, 95% CI [− 0.61, − 0.03]; Figure 2A), but no significant correlation between the magnitude of SPE on RT and IAc (*rho* = −0.04, *p* = 0.82, 95% CI [− 0.36, 0.29]).

Similarly, we examined the relationship separately in the Systole and Diastole conditions (Hypothesis 2). For the Systole condition, the magnitude of SPE on d-prime was significantly negatively correlated with IAc (*rho* = −0.34, *p* = 0.04, 95% CI [− 0.60, − 0.01]; Figure 2B), but not with RT (*rho* = −0.01, *p* = 0.94, 95% CI [−0.34, 0.32]). In the Diastole condition, there was no significant correlation between the magnitude of SPE and IAc (RT: *rho* = −0.08, *p* = 0.63, 95% CI [− 0.40, 0.25]; d-prime: *rho* = −0.27, *p* = 0.11, 95% CI [− 0.55, 0.06]; Figure 2C).

Although the results showed that the magnitude of SPE on d-prime in the Systole condition correlates with IAc, it is unclear how IAc is related to the discriminative processing of self- and other-relevant information. To better understand this, we explored the relationship between d-prime and IAc in the Self and Stranger conditions, respectively. The results showed that there was a significant negative correlation between d-prime in the Self-Systole condition and IAc (*rho* = −0.38, *p* = 0.02, 95% CI [− 0.63, − 0.06]; Figure 2D), but there was no significant correlation between d-prime in the Stranger-Systole condition and IAc (*rho* = 0.15, *p* = 0.39, 95% CI [− 0.19, 0.46]; Figure 2E).

### Effects of the Cardiac Cycle Without Considering IAc

Although this was not our main interest, we conducted paired *t-tests* on the magnitude of SPE on RT and d-prime, respectively, to examine the effects of the cardiac cycle. The results showed that there were no significant differences between the Systole and Diastole conditions for both RT [*t*(35) = 0.74, *p* = 0.47, *d* = 0.12, 95% CI (−0.21, 0.45)] and d-prime [*t*(35) = 0.91, *p* = 0.37, *d* = 0.15, 95% CI (−0.18, 0.48)]. There was no evidence that the cardiac cycle independently affected the magnitude of SPE.

## Discussion

The present study investigated the relationship between the magnitude of SPE measured using the shape-label matching task (Sui et al., 2012) with the presented stimuli based on the participant’s cardiac cycle and IAc measured using the heartbeat counting task (Schandry, 1981). We proposed two possibilities for interoception, namely, that it promotes or inhibits SPE, and hypothesized that (1) IAc is positively or negatively correlated with SPE and (2) the correlation is observed only under conditions where the stimuli are presented during systole in the cardiac cycle where the interoceptive information is accentuated. The results showed a significant negative correlation between the magnitude of SPE on d-prime and IAc. Moreover, a significant correlation was observed only in the Systole condition but not in the Diastole condition. Additionally, IAc was significantly correlated with d-prime in the Self-Systole condition but not in the Stranger-Systole condition. Our results are the first to indicate a relationship between SPE and interoception. More specifically, individuals with higher IAc showed reduced discrimination of temporary self-associated external stimuli when the stimuli were presented coincident with the timing of the cardiac interoceptive information accentuated.

### Preferential Processing of Interoceptive Information by Individuals With Higher IAc, Inhibiting the Discrimination of Temporary Self-Associated External Stimuli

Regarding d-prime, why did individuals with higher IAc show reduced discrimination of temporary self-associated stimuli presented during systole? Both the following two factors may be involved: (1) Preferential processing of internal self-relevant information by individuals with higher IAc, that is, cardiac interoceptive information, over external self-relevant information and (2) enhanced processing of newly self-associated external stimuli was not facilitated by the shape-label matching paradigm situation.

Focusing on (1), we hypothesized that interoception promotes or inhibits SPE, and for inhibition, we considered the findings that IAc was negatively correlated with the strength of illusionary experience in the rubber-hand illusion and enfacement illusion paradigms. In the classic rubber-hand illusion paradigm with visuo-tactile stimulation, it has been reported that individuals with higher IAc are less susceptible to illusions, that is, they are less likely to experience the body-ownership over the fake hands (Tsakiris et al., 2011; Schauder et al., 2015). A similar relationship has been reported in the enfacement illusion paradigm that induces an illusionary experience over the face of the other person (Tajadura-Jiménez and Tsakiris, 2014). These findings showed that individuals with higher IAc are less likely to recognize external bodily stimuli as their own by synchronized visuo-tactile stimulation. Why are individuals with higher IAc are less susceptible to illusions? Individuals with higher IAc are considered to have high precision in their own interoception, and thus their interoceptive information is a salient and reliable source for them (Ainley et al., 2014, 2016). Therefore, it has been indicated that in situations where synchronized visuo-tactile stimulation is presented to induce the illusions that the external bodily stimuli are one’s own (i.e., fake hand or other’s face), individuals with higher IAc are less susceptible to the illusions because they process their own interoceptive information preferentially over exteroceptive information, such as visual observation of synchronized strokes on the external bodily stimuli (Tsakiris et al., 2011; Seth, 2013; Tajadura-Jiménez and Tsakiris, 2014). As for the SPE, it has been proposed that the self-relevant stimuli are processed preferentially because associating with the self increases the saliency of the external stimuli (Sui et al., 2015; Humphreys and Sui, 2016). Considering the results of the present study based on these insights, individuals with higher IAc have high saliency of internal self-relevant information (i.e., interoception), which may inhibit the discriminative processing of external self-relevant stimuli because interoception is processed preferentially over external self-relevant information. This interpretation is also consistent with finding a correlation between IAc and d-prime when the external self-relevant stimuli were presented during systole. No such correlation was observed in the other-relevant stimuli, possibly because the external other-relevant stimuli were not preferentially processed and were not in conflict with the preferential processing of interoceptive information, even in individuals with higher IAc. These results that interoceptive information is involved specifically in self-relevant processing are consistent with those of previous studies (Babo-Rebelo et al., 2016; Qin et al., 2020).

However, considering (2), interoceptive information does not always inhibit the self-relevant processing of external stimuli. In other words, in the shape-label matching paradigm with newly and temporarily associated self-relevant stimuli of the present study, the interoceptive information did not function effectively in associating external stimuli with the self, while interoception can enhance the self-relevant processing of external stimuli when the stimuli and tasks are different. For example, previous studies have reported that observation of fake bodily stimuli pulsating in synchrony with the participant’s heartbeat induced the experience of body-ownership over the fake bodily stimuli (Aspell et al., 2013; Suzuki et al., 2013; Heydrich et al., 2018, 2021). These results suggest that the processing of one’s own interoceptive information is a cue that brings about self-relevance to external stimuli. In such a situation, interoceptive information can be thought of as enhancing the self-relevant processing of external stimuli. As another example, Ambrosini et al. (2019) reported that cardiac interoceptive information enhances the processing of self-face recognition. They presented morphed faces that contained different percentages of the self- and other-face in accordance with the cardiac cycle and asked participants to judge whether they were self- or other-faces. The result showed that participants were faster in judging the morphed faces presented during systole to be their own faces (relative to diastole). Although the details of the process behind this result are unclear, the self-face, which forms associations with the self on a daily basis, carries associations with interoceptive information as well, and when it is processed as external stimuli, the interoceptive information may enhance its processing. In fact, Suzuki et al. (2013) demonstrated that individuals with higher IAc showed a stronger experience of body-ownership by synchronized visuo-tactile stimulation when using virtual hands that have a closer visual appearance to the participant’s real hands, rather than fake hands that are visually very distinct from the appearance of the participant’s real hands, as had been used in the classical rubber-hand illusion studies (e.g., Tsakiris et al., 2011). Unlike the shape-label matching paradigm with newly and temporarily associated self-relevant stimuli, in the situation where interoceptive information serves as a cue for self-relevant processing and/or where the familiar self-relevant stimuli are used, the greater ability to accurately perceive one’s own interoceptive information and accentuation of cardiac interoceptive information during systole could enhance the self-relevant processing of external stimuli.

To summarize, according to our results, individuals with higher IAc showed reduced discrimination of self-relevant stimuli presented during systole in the shape-label matching paradigm with newly self-associated geometric shapes, which can be interpreted as being for two reasons: (1) because individuals with higher IAc preferentially process cardiac interoceptive information over external self-relevant information and (2) because the shape-label matching paradigm with temporary self-associated stimuli that we used did not facilitate enhancement of the self-relevant processing of external stimuli by the cardiac interoceptive information.

### Impact of Interoception on Self-Relevance Judgment Speeds for Newly Self-Associated External Stimuli

Regarding the magnitude of SPE on RT, we found no significant correlation with IAc. Several previous studies have used d-prime and RT as indices of SPE and have demonstrated that self-relevant stimuli are processed preferentially over other-relevant stimuli on both indices, even if different stimuli and sensory modalities are used (e.g., Sui et al., 2012; Frings and Wentura, 2014; Payne et al., 2017). Similar results were replicated in this study using geometric shapes, confirming the robustness of SPE. However, interoception was correlated with SPE on d-prime alone, but not with RT, in the present study. Jiang et al. (2019) examined cultural differences in SPE using both indices, and reported that cultural differences were shown for RT, but not for d-prime. These results, which showed an association with only one of the indices, are not simply due to the speed/accuracy tradeoff in perceptual decision-making, but rather imply that a different cognitive processing is involved in the ability to accurately discriminate the stimuli and the speed of making the decision. Interoception may have a specific effect only on discriminative processing of temporary self-associated external stimuli.

### Limitations

The following limitations are to be considered. The first is regarding the causal relationship between interoception and SPE. To get closer to the elucidation of the causal relationship, we presented stimuli based on the participant’s cardiac cycle in the shape-label matching paradigm. As a result, we observed a correlation between IAc and SPE only in the Systole condition without the main effect of cardiac cycle on SPE. Thus, our conclusions are based on individual differences and we infer the causal relationship between interoception and SPE based on the assumption that individuals with higher IAc would be more affected by cardiac afferent information. To identify differences in IAc as the cause of the magnitude of SPE, it is necessary to confirm whether the SPE changes as a result of changing IAc through an intervention. Considering that IAc could be improved by non-invasive vagus nerve stimulation (Villani et al., 2019) and physical activity (Quadt et al., 2021), further research would be able to investigate a more direct causality by using these methods.

Second, our findings are limited to the cardiac axis, which is part of interoception. While the cardiac axis has been the subject of most interoceptive studies, different bodily axes may be available as well. For example, several tasks have been used to measure interoception in the respiratory and gastric axes, and individual differences in how we process them have been reported (Garfinkel et al., 2016; van Dyck et al., 2016). Garfinkel et al. (2016) examined the relationship between cardiac and respiratory interoception and reported that although no relationship was found in interoceptive accuracy, a positive relationship was found for interoceptive awareness, which is metacognition in one’s own interoceptive ability (Garfinkel et al., 2015). Additionally, regarding the relationship between cardiac and gastric interoception, van Dyck et al. (2016) found that cardiac interoceptive accuracy and the percentage of satiation to maximum fullness measured by the water load test were not related. Although it is not easy to conclude, as different methods are applied, our interoceptive processing is specific to each axis and may have different impacts on our cognitive processing.

Third, the validity of the heartbeat counting task (Schandry, 1981) used to measure IAc can be called into question. This task is commonly used in the field of interoceptive studies, including the studies (classical rubber-hand illusion: Tsakiris et al., 2011; Schauder et al., 2015; enfacement illusion: Tajadura-Jiménez and Tsakiris, 2014) that we referenced to discuss the relationship between interoception and SPE because it is non-invasive and easy to perform. However, it has been argued that the performance of the task is affected by non-interoceptive factors (Ring et al., 2015; Zamariola et al., 2018; Ainley et al., 2020; Corneille et al., 2020; Zimprich et al., 2020). To reduce the effect of knowledge about heartbeats on performance (Ring et al., 2015), we referred to Desmedt et al. (2018) and instructed the participants to count only their perceived heartbeats and not to guess the heartbeats based on their knowledge; however, response bias may have affected this (Corneille et al., 2020).

Finally, how interoception impacted the SPE in this study was unclear. Previous studies have shown that associating stimuli with the self modulates multiple stages of information processing, including attention, memory, and decision-making (Cunningham and Turk, 2017; Sui and Humphreys, 2017). For the emergence of SPE, although attention has been proposed to play an important role (Sui et al., 2015; Humphreys and Sui, 2016), memory has been reported to influence task performance as well (Yin et al., 2019; Caughey et al., 2021). In this study, we observed that individuals with higher IAc showed reduced discrimination of temporarily self-associated stimuli when stimuli were presented during systole, which is the timing at which cardiac interoceptive information is accentuated. However, multiple stages of information processing are involved in this discrimination, and it is unclear which of these processes were inhibited by interoceptive processing.

## Conclusion

In the present study, we demonstrated that (1) IAc is negatively correlated with SPE and (2) the correlation was observed only when the stimuli were presented during cardiac systole. Additionally, (3) IAc was negatively correlated with the processing of self-relevant rather than other-relevant stimuli. These results can be interpreted to reflect that individuals with higher IAc preferentially process cardiac interoceptive information over external self-relevant information in situations using the shape-label matching paradigm with newly self-associated stimuli. For an integrated understanding with the findings of previous studies, we also discussed the possibility that interoceptive information may enhance the processing of external self-relevant stimuli when using experimental paradigms other than SPE and familiarity-rich self-relevant stimuli. Our study shed more light on the involvement of interoception in the preferential processing of external self-relevant information.

## Data Availability Statement

The raw data supporting the conclusions of this article will be made available by the authors, without undue reservation.

## Ethics Statement

The studies involving human participants were reviewed and approved by the Ethical Committee of the Graduate School of Education, Hiroshima University. The patients/participants provided their written informed consent to participate in this study.

## Author Contributions

TH and TN contributed to the design of the study. TH conducted the study, analyzed the data, and wrote the first draft. All authors contributed to the article and approved the submitted version.

## Funding

This research was supported by the Center of Innovation Program of the Japan Science and Technology Agency (JST) JPMJCE1311 and JPMJCA2208.

## Conflict of Interest

The authors declare that the research was conducted in the absence of any commercial or financial relationships that could be construed as a potential conflict of interest.

## Publisher’s Note

All claims expressed in this article are solely those of the authors and do not necessarily represent those of their affiliated organizations, or those of the publisher, the editors and the reviewers. Any product that may be evaluated in this article, or claim that may be made by its manufacturer, is not guaranteed or endorsed by the publisher.

## References

[ref1] Ainley V. Apps M. A. J. Fotopoulou A. Tsakiris M. (2016). ‘Bodily precision’: A predictive coding account of individual differences in interoceptive accuracy. Philos. Trans. R. Soc. Lond. Ser. B Biol. Sci. 371:20160003. doi: 10.1098/rstb.2016.0003, PMID: 28080962 PMC5062093

[ref2] Ainley V. Brass M. Tsakiris M. (2014). Heartfelt imitation: high interoceptive awareness is linked to greater automatic imitation. Neuropsychologia 60, 21–28. doi: 10.1016/j.neuropsychologia.2014.05.010, PMID: 24874609

[ref3] Ainley V. Tsakiris M. Pollatos O. Schulz A. Herbert B. M. (2020). Comment on “Zamariola et al. (2018), Interoceptive accuracy scores are problematic: evidence from simple bivariate correlations”—the empirical data base, the conceptual reasoning and the analysis behind this statement are misconceived and do not support the authors’ conclusions. Biol. Psychol. 152:107870. doi: 10.1016/j.biopsycho.2020.107870 32061687

[ref4] Ambrosini E. Finotti G. Azevedo R. T. Tsakiris M. Ferri F. (2019). Seeing myself through my heart: cortical processing of a single heartbeat speeds up self-face recognition. Biol. Psychol. 144, 64–73. doi: 10.1016/j.biopsycho.2019.03.006, PMID: 30890454

[ref5] Aspell J. E. Heydrich L. Marillier G. Lavanchy T. Herbelin B. Blanke O. (2013). Turning body and self inside out: visualized heartbeats alter bodily self-consciousness and tactile perception. Psychol. Sci. 24, 2445–2453. doi: 10.1177/0956797613498395, PMID: 24104506

[ref6] Babo-Rebelo M. Richter C. G. Tallon-Baudry C. (2016). Neural responses to heartbeats in the default network encode the self in spontaneous thoughts. J. Neurosci. 36, 7829–7840. doi: 10.1523/JNEUROSCI.0262-16.2016, PMID: 27466329 PMC4961773

[ref7] Baranauskas M. Grabauskaitė A. Griškova-Bulanova I. Lataitytė-Šimkevičienė B. Stanikūnas R. (2021). Heartbeat evoked potentials (HEP) capture brain activity affecting subsequent heartbeat. Biomed. Signal Process Control 68:102731. doi: 10.1016/j.bspc.2021.102731

[ref8] Berntson G. G. Khalsa S. S. (2021). Neural circuits of interoception. Trends Neurosci. 44, 17–28. doi: 10.1016/j.tins.2020.09.011, PMID: 33378653 PMC8054704

[ref9] Botvinick M. Cohen J. (1998). Rubber hands ‘feel’ touch that eyes see. Nature 391:756. doi: 10.1038/35784, PMID: 9486643

[ref10] Caughey S. Falbén J. K. Tsamadi D. Persson L. M. Golubickis M. Neil Macrae C. (2021). Self-prioritization during stimulus processing is not obligatory. Psychol. Res. 85, 503–508. doi: 10.1007/s00426-019-01283-2, PMID: 31919569 PMC7900024

[ref11] Corneille O. Desmedt O. Zamariola G. Luminet O. Maurage P. (2020). A heartfelt response to Zimprich et al. (2020), and Ainley et al. (2020)‘s commentaries: acknowledging issues with the HCT would benefit interoception research. Biol. Psychol. 152:107869. doi: 10.1016/j.biopsycho.2020.107869, PMID: 32061686

[ref12] Craig A. D. (2009). How do you feel — now? The anterior insula and human awareness. Nat. Rev. Neurosci. 10, 59–70. doi: 10.1038/nrn2555 19096369

[ref13] Critchley H. D. Garfinkel S. N. (2018). The influence of physiological signals on cognition. Curr. Opin. Behav. Sci. 19, 13–18. doi: 10.1016/j.cobeha.2017.08.014

[ref14] Cunningham S. J. Turk D. J. (2017). Editorial: a review of self-processing biases in cognition. Q. J. Exp. Psychol. (Hove) 70, 987–995. doi: 10.1080/17470218.2016.1276609, PMID: 28059625

[ref15] Desmedt O. Luminet O. Corneille O. (2018). The heartbeat counting task largely involves non-interoceptive processes: evidence from both the original and an adapted counting task. Biol. Psychol. 138, 185–188. doi: 10.1016/j.biopsycho.2018.09.004, PMID: 30218689

[ref16] Frings C. Wentura D. (2014). Self-priorization processes in action and perception. J. Exp. Psychol. Hum. Percept. Perform. 40, 1737–1740. doi: 10.1037/a0037376, PMID: 24999614

[ref17] Garfinkel S. N. Critchley H. D. (2016). Threat and the body: how the heart supports fear processing. Trends Cogn. Sci. 20, 34–46. doi: 10.1016/j.tics.2015.10.005, PMID: 26628111

[ref18] Garfinkel S. N. Manassei M. F. Hamilton-Fletcher G. den Bosch Y. I. Critchley H. D. Engels M. (2016). Interoceptive dimensions across cardiac and respiratory axes. Philos. Trans. R. Soc. B 371:20160014. doi: 10.1098/rstb.2016.0014, PMID: 28080971 PMC5062102

[ref19] Garfinkel S. N. Minati L. Gray M. A. Seth A. K. Dolan R. J. Critchley H. D. (2014). Fear from the heart: sensitivity to fear stimuli depends on individual heartbeats. J. Neurosci. 34, 6573–6582. doi: 10.1523/JNEUROSCI.3507-13.2014, PMID: 24806682 PMC4012313

[ref20] Garfinkel S. N. Seth A. K. Barrett A. B. Suzuki K. Critchley H. D. (2015). Knowing your own heart: distinguishing interoceptive accuracy from interoceptive awareness. Biol. Psychol. 104, 65–74. doi: 10.1016/j.biopsycho.2014.11.004, PMID: 25451381

[ref21] Golubickis M. Falben J. K. Sahraie A. Visokomogilski A. Cunningham W. A. Sui J.. (2017). Self-prioritization and perceptual matching: the effects of temporal construal. Mem. Cogn. 45, 1223–1239. doi: 10.3758/s13421-017-0722-3, PMID: 28593461 PMC5605582

[ref22] Gray M. A. Beacher F. D. Minati L. Nagai Y. Kemp A. H. Harrison N. A.. (2012). Emotional appraisal is influenced by cardiac afferent information. Emotion 12, 180–191. doi: 10.1037/a0025083, PMID: 21988743

[ref23] Heydrich L. Aspell J. E. Marillier G. Lavanchy T. Herbelin B. Blanke O. (2018). Cardio-visual full body illusion alters bodily self-consciousness and tactile processing in somatosensory cortex. Sci. Rep. 8:9230. doi: 10.1038/s41598-018-27698-2, PMID: 29915337 PMC6006256

[ref24] Heydrich L. Walker F. Blättler L. Herbelin B. Blanke O. Aspell J. E. (2021). Interoception and empathy impact perspective taking. Front. Psychol. 11:599429. doi: 10.3389/fpsyg.2020.599429, PMID: 33536971 PMC7848222

[ref25] Humphreys G. W. Sui J. (2016). Attentional control and the self: the self-attention network (SAN). Cogn. Neurosci. 7, 5–17. doi: 10.1080/17588928.2015.1044427, PMID: 25945926

[ref26] Jiang M. Wong S. K. M. Chung H. K. S. Sun Y. Hsiao J. H. Sui J.. (2019). Cultural orientation of self-bias in perceptual matching. Front. Psychol. 10:1469. doi: 10.3389/fpsyg.2019.01469, PMID: 31316430 PMC6610885

[ref27] Kim H. Florack A. (2021). Immediate self-information is prioritized over expanded self-information across temporal, social, spatial, and probability domains. Q. J. Exp. Psychol. (Hove) 74, 1615–1630. doi: 10.1177/17470218211004208, PMID: 33719761 PMC8358571

[ref28] Moray N. (1959). Attention in dichotic listening: affective cues and the influence of instructions. Q. J. Exp. Psychol. (Hove) 11, 56–60. doi: 10.1080/17470215908416289

[ref29] Payne S. Tsakiris M. Maister L. (2017). Can the self become another? Investigating the effects of self-association with a new facial identity. Q. J. Exp. Psychol. (Hove) 70, 1085–1097. doi: 10.1080/17470218.2015.1137329, PMID: 26822152

[ref30] Pronin E. Olivola C. Y. Kennedy K. A. (2008). Doing unto future selves as you would do unto others: psychological distance and decision making. Personal. Soc. Psychol. Bull. 34, 224–236. doi: 10.1177/0146167207310023, PMID: 18156588

[ref31] Pronin E. Ross L. (2006). Temporal differences in trait self-ascription: when the self is seen as an other. J. Pers. Soc. Psychol. 90, 197–209. doi: 10.1037/0022-3514.90.2.197, PMID: 16536646

[ref32] Qin P. Wang M. Northoff G. (2020). Linking bodily, environmental and mental states in the self—a three-level model based on a meta-analysis. Neurosci. Biobehav. Rev. 115, 77–95. doi: 10.1016/j.neubiorev.2020.05.004, PMID: 32492474

[ref33] Quadt L. Garfinkel S. N. Mulcahy J. S. Larsson D. E. Silva M. Jones A.-M.. (2021). Interoceptive training to target anxiety in autistic adults (ADIE): a single-center, superiority randomized controlled trial. eClinicalMedicine 39:101042. doi: 10.1016/j.eclinm.2021.101042, PMID: 34401684 PMC8350004

[ref34] Ring C. Brener J. Knapp K. Mailloux J. (2015). Effects of heartbeat feedback on beliefs about heart rate and heartbeat counting: a cautionary tale about interoceptive awareness. Biol. Psychol. 104, 193–198. doi: 10.1016/j.biopsycho.2014.12.010, PMID: 25553874

[ref35] Schäfer S. Wesslein A.-K. Spence C. Wentura D. Frings C. (2016). Self-prioritization in vision, audition, and touch. Exp. Brain Res. 234, 2141–2150. doi: 10.1007/s00221-016-4616-6, PMID: 26979440

[ref36] Schandry R. (1981). Heart beat perception and emotional experience. Psychophysiology 18, 483–488. doi: 10.1111/j.1469-8986.1981.tb02486.x, PMID: 7267933

[ref37] Schauder K. B. Mash L. E. Bryant L. K. Cascio C. J. (2015). Interoceptive ability and body awareness in autism spectrum disorder. J. Exp. Child Psychol. 131, 193–200. doi: 10.1016/j.jecp.2014.11.002, PMID: 25498876 PMC4303499

[ref38] Sel A. Sui J. Shepherd J. Humphreys G. (2019). Self-association and attentional processing regarding perceptually salient items. Rev. Phil. Psych. 10, 735–746. doi: 10.1007/s13164-018-0430-3

[ref39] Seth A. K. (2013). Interoceptive inference, emotion, and the embodied self. Trends Cogn. Sci. 17, 565–573. doi: 10.1016/j.tics.2013.09.007, PMID: 24126130

[ref001] Snodgrass J. G. Corwin J. (1988). Pragmatics of measuring recognition memory: applications to dementia and amnesia. J. Exp. Psychol. Gen. 117, 34–50. 2966230 10.1037//0096-3445.117.1.34

[ref40] Sui J. Gu X. (2017). Self as object: emerging trends in self research. Trends Neurosci. 40, 643–653. doi: 10.1016/j.tins.2017.09.002, PMID: 28988827

[ref41] Sui J. He X. Humphreys G. W. (2012). Perceptual effects of social salience: evidence from self-prioritization effects on perceptual matching. J. Exp. Psychol. Hum. Percept. Perform. 38, 1105–1117. doi: 10.1037/a0029792, PMID: 22963229

[ref42] Sui J. Humphreys G. W. (2015). The integrative self: how self-reference integrates perception and memory. Trends Cogn. Sci. 19, 719–728. doi: 10.1016/j.tics.2015.08.015, PMID: 26447060

[ref43] Sui J. Humphreys G. W. (2017). The ubiquitous self: what the properties of self-bias tell us about the self. Ann. N. Y. Acad. Sci. 1396, 222–235. doi: 10.1111/nyas.13197, PMID: 27918835 PMC6029667

[ref44] Sui J. Liu M. Mevorach C. Humphreys G. W. (2015). The salient self: The left intraparietal sulcus responds to social as well as perceptual-salience after self-association. Cereb. Cortex 25, 1060–1068. doi: 10.1093/cercor/bht302, PMID: 24165832

[ref45] Suzuki K. Garfinkel S. N. Critchley H. D. Seth A. K. (2013). Multisensory integration across exteroceptive and interoceptive domains modulates self-experience in the rubber-hand illusion. Neuropsychologia 51, 2909–2917. doi: 10.1016/j.neuropsychologia.2013.08.014, PMID: 23993906

[ref46] Tajadura-Jiménez A. Tsakiris M. (2014). Balancing the “inner” and the “outer” self: interoceptive sensitivity modulates self-other boundaries. J. Exp. Psychol. Gen. 143, 736–744. doi: 10.1037/a0033171, PMID: 23750913 PMC3848898

[ref47] Tsakiris M. Critchley H. (2016). Interoception beyond homeostasis: affect, cognition and mental health. Philos. Trans. R. Soc. Lond. Ser. B Biol. Sci. 371:20160002. doi: 10.1098/rstb.2016.0002, PMID: 28080961 PMC5062092

[ref48] Tsakiris M. Jiménez A. T. Costantini M. (2011). Just a heartbeat away from one’s body: interoceptive sensitivity predicts malleability of body-representations. Proc. R. Soc. B Biol. Sci. 278, 2470–2476. doi: 10.1098/rspb.2010.2547, PMID: 21208964 PMC3125630

[ref49] van Dyck Z. Vögele C. Blechert J. Lutz A. P. C. Schulz A. Herbert B. M. (2016). The water load test as a measure of gastric interoception: development of a two-stage protocol and application to a healthy female population. PLoS One 11:e0163574. doi: 10.1371/journal.pone.0163574, PMID: 27657528 PMC5033375

[ref50] Verplanken B. Sui J. (2019). Habit and identity: behavioral, cognitive, affective, and motivational facets of an integrated self. Front. Psychol. 10:1504. doi: 10.3389/fpsyg.2019.01504, PMID: 31354563 PMC6635880

[ref51] Villani V. Tsakiris M. Azevedo R. T. (2019). Transcutaneous vagus nerve stimulation improves interoceptive accuracy. Neuropsychologia 134:107201. doi: 10.1016/j.neuropsychologia.2019.107201, PMID: 31562863

[ref52] von Mohr M. Finotti G. Villani V. Tsakiris M. (2021). Taking the pulse of social cognition: cardiac afferent activity and interoceptive accuracy modulate emotional egocentricity bias. Cortex 145, 327–340. doi: 10.1016/j.cortex.2021.10.004, PMID: 34794068

[ref53] Yin S. Sui J. Chiu Y. C. Chen A. Egner T. (2019). Automatic prioritization of self-referential stimuli in working memory. Psychol. Sci. 30, 415–423. doi: 10.1177/0956797618818483, PMID: 30653399

[ref54] Zamariola G. Maurage P. Luminet O. Corneille O. (2018). Interoceptive accuracy scores from the heartbeat counting task are problematic: evidence from simple bivariate correlations. Biol. Psychol. 137, 12–17. doi: 10.1016/j.biopsycho.2018.06.006, PMID: 29944964

[ref55] Zimprich D. Nusser L. Pollatos O. (2020, 2018). Are interoceptive accuracy scores from the heartbeat counting task problematic? A comment on Zamariola et al. Biol. Psychol. 152:107868. doi: 10.1016/j.biopsycho.2020.107868 32097681

